# Gestational diabetes mellitus increased the number of dopaminergic neurons in the olfactory bulb of rat offspring

**DOI:** 10.22038/IJBMS.2023.71300.15488

**Published:** 2023

**Authors:** Zahra Nazari, Khadijeh Bahrehbar, Soraya Ghafari, Mohammad Jafar Golalipour

**Affiliations:** 1 Department of Biology, Faculty of Sciences, Golestan University, Gorgan, Iran; 2 Department of Biology, Faculty of Basic Sciences, Yasuj University, Yasuj, Iran; 3 Department of Stem Cells and Developmental Biology, Cell Science Research Center, Royan Institute for Stem Cell Biology and Technology, ACECR, Tehran, Iran; 4 Congenital Malformations Research Center, Golestan University of Medical Sciences, Gorgan, Iran

**Keywords:** Dopaminergic neurons, Gestational diabetes, Olfactory bulb, Streptozotocin, Tyrosine hydroxylase

## Abstract

**Objective(s):**

Gestational Diabetes Mellitus (GDM) is the most common metabolic complication of pregnancy that causes central nervous system and olfactory dysfunction in the offspring. It has been demonstrated that dopamine modulates several aspects of olfactory information processing in vertebrates.

**Materials and Methods:**

In this study, we investigated the effect of gestational diabetes on the expression of the Dopamine (DA) metabolism genes, tyrosine hydroxylase (TH), and dopa decarboxylase (DDC) in the olfactory bulb (OB) tissue of rats’ offspring. Female Wistar rats were divided into a control group which received citrate buffer and the diabetic group which received 45 mg/kg of streptozotocin (STZ) on day 0 of gestation. Fasting blood glucose levels were measured before and 72 hr after injection. OB tissues of adult offspring were isolated, and TH-positive cells were counted by immunofluorescence staining. Also, TH and DDC expressions were analyzed by qRT- PCR and western blot.

**Results:**

The data showed that gestational diabetes could cause up-regulation of TH (*P*<0.01) and DDC (P<0.05) in the OB tissue of offspring. Furthermore, our results showed that GDM causes a significant increase in TH and DDC protein levels in the OB tissues of offspring. Immunohistochemistry showed a significant increase in the number of TH-positive cells in the offspring of diabetic mothers (*P*<0.05).

**Conclusion:**

This study showed that gestational diabetes could cause an increase in TH and DDC gene expression in the OB tissue in the offspring, which may be correlated with reduced olfactory sensitivity.

## Introduction

Diabetes mellitus (DM) is one of the most chronic metabolic disorders characterized by hyperglycemia and metabolic changes in lipids, sugars, and proteins (1). The most common types of diabetes include type 1 diabetes (T1D), type 2 diabetes (T2D), and gestational diabetes Mellitus (GDM) (2). GDM is impaired glucose tolerance that develops during pregnancy and affects approximately 7 % of all pregnant women (3). GDM is associated with maternal and fetal risks (4). Our group previously reported that GDM affects the development of various organs, such as the heart, brain, and pancreas, in rodent offspring (5-9).

Olfaction is essential for our interaction with the environment. In rodents, olfaction plays a significant role in mate choice, maternal behavior, and food detection (10). Diabetes affects the olfactory system, and several studies indicated that the prevalence of olfactory dysfunction increased in these patients (11-13). In recent years, many clinical and experimental studies have been performed on the effect of maternal diabetes on the central nervous system (CNS) and peripheral nervous system (PNS) development. The CNS malformation risk is approximately 15.5 times higher in the children of diabetics mothers (14, 15). It has been reported that the offspring of diabetic rats have high levels of dopamine and norepinephrine in the hypothalamus and increased dopamine, norepinephrine, and serotonin at the caudate nucleus (16).

The olfactory bulb (OB) is a brain region involved in the early processing of olfactory information. The OB tissue consists of several layers, such as the olfactory nerve layer, glomerular layer (GL), external plexiform layer (EPL), mitral layer (MCL), and internal plexiform layer (IPL) (17, 18). The GL has several interneurons, such as the GABAergic and dopaminergic neurons, which regulate olfactory processing (19, 20). Dopaminergic neurons of the OB represent the most numerous endogenous dopaminergic cells in the forebrain (21). The majority of dopaminergic neurons appeared in the glomerular layer that expresses dopaminergic markers: dopa decarboxylase (DDC), also known as aromatic-L-amino acid decarboxylase (AADC) and Tyrosine hydroxylase (TH), which are the rate-limiting enzyme of dopamine synthesis (22, 23).

Several post-mortem studies showed that the number of dopaminergic cells is inversely related to olfactory power. Aging and also pathological condition such as Parkinson’s disease are associated with a decrease in olfactory sensitivity. On the other hand, studies reported that the number of dopaminergic cells is increased in these people. Therefore, increased dopaminergic neurons in the OB tissue might be related to decreased olfactory sensitivity (24)**. **In this study, we established the streptozotocin-induced rat model of gestational diabetes, then the expression of TH and DDC in the OB tissue of male offspring of gestational diabetes (OGD) and control rats evaluated by immunofluorescence, qRT-PCR, and western blotting analysis.

## Materials and Methods

***Experimental animal***

This experimental study was performed on 24 mature female Wistar rats (200~250 g, 8**–**10 weeks old). The rats were housed under a 12-12 hr light-dark cycle and had free access to food and tap water. All animal experiments were approved by the ethical committee of Golestan University of Medical Sciences (Ethical code: IR.goums.REC.1395.192).

***Establishment of the streptozotocin-induced rat model of diabetes***

Female rats were separately placed with proven breeder male Wistar rats (200~250 g, 8**–**10 weeks old) overnight for breeding. The vaginal plug was monitored daily, and the day of vaginal plug detection was considered day 0 of pregnancy. Pregnant rats were randomly divided into two control and diabetic groups. We dissolved STZ in 0.1 M citrate buffer (pH 4.5) to induce diabetes in rats. Based on previous protocols, the control group received citrate buffer (STZ solvent), and the diabetic group intraperitoneally received 45 mg/kg of STZ (Sigma- Aldrich, USA) on day 0 of gestation (D0) (25-27). In order to confirm the successful establishment of GDM in the rat model, the blood glucose level was checked daily before and 72 hr after STZ injection using a glucometer (Optium Xceed, UK). In the STZ-injected group, rats with a glucose concentration exceeding 120 mg/dl were considered as the GDM model. The rats were allowed to deliver spontaneously, and the male pups were kept until adulthood. Since gender affects olfactory sensitivity, to obtain accurate and reliable results, only male offspring were evaluated in this research**.** Six male offspring from each diabetic and control mother at the age of 15 weeks were selected. Animals were anesthetized by intraperitoneal injection of ketamine (100 mg/kg) and xylazine (10 mg/kg), and then OB tissues were extracted and used for immunofluorescence analysis, qRT-PCR, and western blotting.

***Gene expression analysis***

Quantitative reverse transcriptase–polymerase chain reaction (qRT-PCR) was performed to evaluate TH and DDC gene levels in OB tissue. Total RNA was isolated and purified with a total RNA purification kit (Takara). Then total RNA was reverse transcribed to cDNA using the Easy cDNA Synthesize (Takara) kit according to the producer’s protocol. Based on previously published protocols, qRT-PCR reactions were performed using SYBR Green Master Mix (Jena Bioscience) in an ABI 7300 Real-Time PCR System (28, 29). The fold change for each gene was normalized to GAPDH. Gene expression was determined using the 2^-ΔΔCT^ method. The sequences of primers are listed in Table 1. 

***Western blot analysis***

The level of TH and DDC protein in the OB tissue was assessed by western blot. OB tissue of six offspring from each group was separated on ice. Total protein was extracted by 500 lL of NP40 lysis buffer supplemented with 1mM PMSF and 500 lL of protease inhibitor cocktail. The supernatant protein content was measured using a Bradford assay. We performed a western blot according to previously published protocols using DDC (Invitrogen, CL2962) and TH (Sigma-Aldrich, SAB4300675) primary antibodies (30). Briefly, 35 μg of protein from each sample was separated on SDS-PAGE and transferred onto nitrocellulose membranes. The blots were incubated in a blocking buffer for 1 hr at room temperature. Then, the membranes were incubated overnight at 4 °C with DDC and TH primary antibodies (1:1000). β-actin was used as an internal control (Abcam, ab8226). Finally, an Immune-blot assay kit (Bio-Rad, USA) was used to visualize the protein bands. After scanning the blots, they were quantified using the ImageJ Software package. 

***Immunofluorescence***

In addition to examining mRNA expression and protein level of TH, we performed immunofluorescence staining based on a previous study (31). Briefly, we examined the number of TH-positive neurons in the OB tissue of both groups. For this aim, the OB tissues from six control and six OGD samples were fixed in 4% paraformaldehyde (Sigma-Aldrich), embedded in paraffin, and cut into 6 μm sections by microtome (MicromHm325, Thermo Scientific). For antigen retrieval, sections were boiled in citrate buffer in an oven for 20 min. Then, they were permeated with 0.1% Triton X-100 (Sigma, USA) for 10 min and blocked with BSA for 30 min at room temperature. The samples were washed twice with PBS and kept in a blocking solution for 30 min and then incubated with primary antibody anti-TH (ab137869, 1:200) overnight at 4 °C. After washing with PBS, the samples were incubated with FITC-conjugated secondary antibody (65-6111, Thermo Scientific) in the dark for 60 min at room temperature. For imaging, the slides were observed under a fluorescent microscope (Olympus BX51), and the number of TH-positive cells was counted. TH-positive neurons were counted in 6 slices from one OB tissue, and the average across all sections was used to represent the number of dopaminergic neurons. TH-positive neurons were counted per 2 mm2 random areas of the glomerular layer. Counterstaining of the nucleus was done using DAPI staining to quantify the TH-positive neurons in the glomerular layer. Cell counting was conducted in the ImageJ Software package.

***Statistical analysis***

The data were analyzed by SPSS software (v.18). All experiments were conducted in at least three independent repeats. T-test analysis of variance was used to determine significant differences among groups with Tukey’s *post hoc* test. Mean data in each group were compared using a t-test. All data are shown as mean ± standard error of the mean, and *P*<0.05 was considered statistically significant.

## Results

***Serum glucose level***

**Figure 1:**
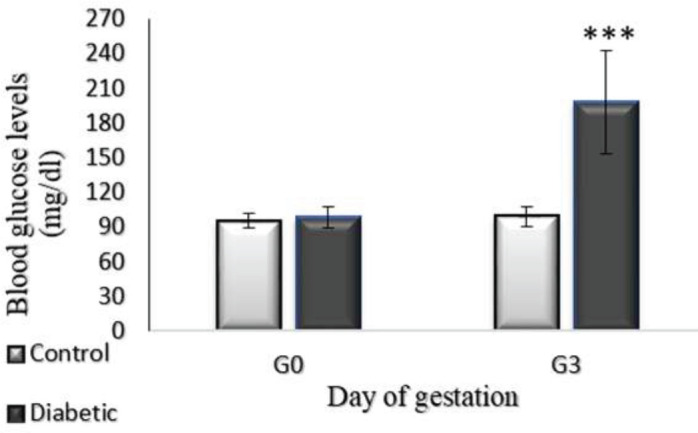
Changes in serum glucose levels of pregnant rats on day 0 of pregnancy (G0) and day 3 of pregnancy (G3). Data are expressed as mean±SEM (n=6, *** *P*<0.001)

Serum glucose levels were measured by a glucometer in the STZ-induced diabetic group and control group before and 72 hr after the injection of STZ. The results showed that fasting blood glucose concentration was significantly increased in GDM rats 72 hr after STZ injection. (Figure 1, *P*<0.001).

***Effects of GDM on mRNA and protein levels of Dopamine-related genes in OB***

**Figure 2:**
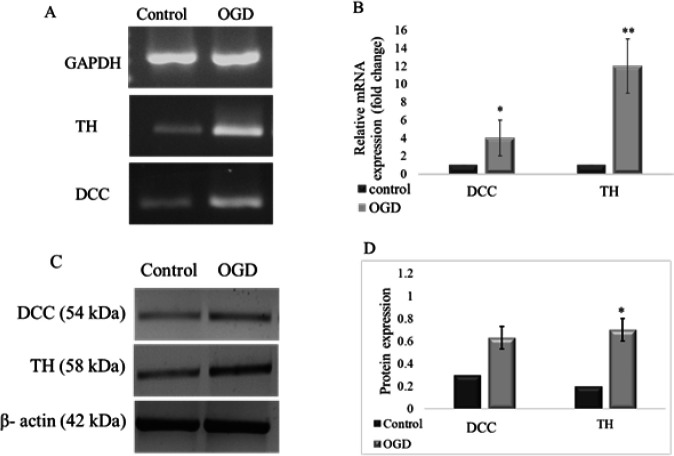
QRT- PCR and immunoblotting analysis of TH and DDC levels in the OB tissue of offspring from diabetic and control groups

We assessed whether the expression levels of dopamine-related genes** (***TH* and *DDC***) **were affected by gestational diabetes in adult offspring. QRT-PCR results of *TH* and *DDC* expression are shown in Figure 2 (A, B). The expression level of the *DDC* gene in isolated OB tissue of the OGD group was significantly higher than those of controls (*P<0.05), indicating that the expression of these genes was disturbed by GDM. Also, the *TH* mRNA levels analysis showed a significant increase in the OGD group compared to the control group (** *P*<0.01). 

To further confirm whether GDM increases the protein level of TH and DDC in the OB tissue of offspring, we performed a western blot assessment. Both proteins in the OGD samples showed an increased band intensity in nitrocellulose membrane images (Figure 2C), which correlates with and confirms our results observed in the qRT-PCR analysis. Quantitative analysis of western blotting bands by ImageJ software showed that GDM causes a significant increase in the expression level of TH protein in the OGD samples (* *P*<0.05, Figure 2D). In this experiment, β- actin protein was used as an internal control.

***Effects of GDM on the number of dopaminergic neurons in OB***

**Figure 3:**
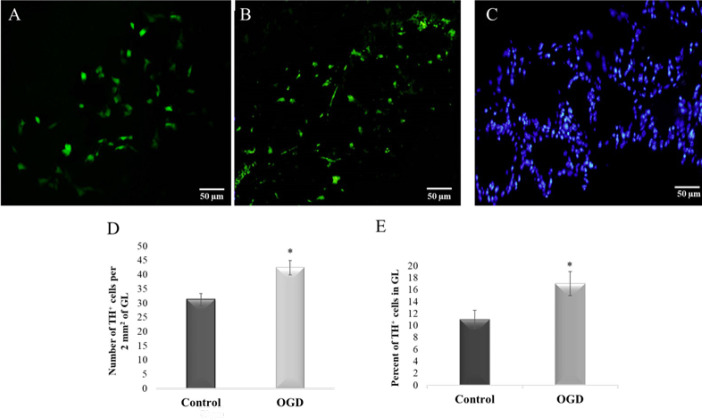
Typical examples of immunofluorescence staining of OB using FITC-labelled TH antibody in both control and OGD group

TH immunostaining in OB tissue from control and OGD rats indicated that in OGD samples, staining was greatly increased in the GL layer (Figure 3 A, B). Statistical analysis showed a significant increase in TH-positive cells in the OGD samples compared to controls (*P<0.05, Figure 3D, 42.4±2.5 cells vs. 31.2±2.1). Our data from counterstaining the GL layer using DAPI staining showed that the percent of TH-positive cells in control and OGD samples are 11% and 17%, respectively (*P<0.05 Figure 3E).

**Table 1:** Primers for qRT-PCR analysis

**Gene**	**Forward primer (5'- 3')**	**Reverse primer (5'- 3')**
GAPDH	ACAGCAACAGGGTGGTG	TTTGAGGGTGCAGCGAACTT
DDC	CCTACTGGCTGCTCGGACTA	TTTCTACGGAGGAATGTGCC
TH	CGGGCTATGTAAACAGAATGG	CACAGGCTGGTAGGTTTGATC

DDC: Dopa decarboxylase; GAPDH: Glyceraldehyde 3-phosphate dehydrogenase; qRT-PCR: Quantitative real-time polymerase chain reaction; TH: Tyrosine hydroxylase

A) Agarose gel electrophoresis of PCR products following real-time SYBR green amplification. B) Data analysis of TH and DDC mRNA expression shows a significant increase for both genes in the OB tissue of the OGD group compared to controls. C) Nitrocellulose membrane images obtained from immunoblotting of TH, DDC, and β- actin proteins in the OB tissue of the 15-week-old OGD and control groups. Monoclonal antibody β-actin was used to analyze the expression of a housekeeping gene product. D) Quantified data of the western blotting results. The level of both proteins increased in the OB tissue of the OGD group compared to the control group, which was significant for TH. Data are expressed as mean±SEM (n=6, * *P*<0.05, ** *P*<0.01).

DDC: Dopa decarboxylase; qRT-PCR: Quantitative real-time polymerase chain reaction; TH: Tyrosine hydroxylase; OB: olfactory bulb

Photomicrograph showing a section of the GL layer of the OB tissue in control (A) and OGD (B) samples. The dopaminergic cells' immunopositivity for TH is shown in green. (C) Photomicrograph shows a counterstaining of the GL layer with DAPI staining in the OGD group. (D) Result of cell counting showed a significant increase in the number of TH staining in the GL layer of OGD samples. (E) Percent of TH-positive cells in control and OGD group. Data are expressed as mean ± standard error of the mean (n=6, *P<0.05), Scale bar =50 μm.

OGD: Offspring of gestational diabetes; OB: OB: Olfactory bulb; TH: Tyrosine hydroxylase; GL: Glomerular layer

## Discussion

In humans, there is a relationship between olfactory dysfunction and diseases such as Alzheimer’s, Parkinson’s, schizophrenia, and depression. Furthermore, based on recent studies, olfactory impairment is related to diabetes (32). Increasing evidence suggests a link between diabetes and olfactory dysfunction or a reduced ability to detect or identify odors. While the exact mechanism behind this link is not yet fully understood, several studies have shown that people with diabetes are more likely to experience olfactory dysfunction compared to those without the condition. Research has also suggested that olfactory dysfunction may be an early sign of diabetes, even before other symptoms, such as high blood sugar levels, become apparent. In one study, researchers found that people with an impaired sense of smell were more likely to develop diabetes over a five-year period, even after accounting for other risk factors such as age, sex, and body mass index (13, 33, 34). Possible mechanisms of olfactory dysfunction associated with diabetes include neurodegeneration, glucotoxicity, insulin resistance, and vascular dysfunction (32, 35). 

The OB tissue is the first central station for olfactory processing (36). Within the OB tissue, dopaminergic neurons that are identified by the expression of TH have been reported almost only in the glomerular layer (32, 37). As we know, aging is correlated with a decline in olfactory performance. Alizadeh *et al*. showed that dopaminergic markers TH and DDC expression increases with age. Furthermore, their results indicated that the number of dopaminergic neurons in the OB tissue is higher in men than in women (37). Because healthy females have better olfactory performance than males, there may be a negative relationship between the number of dopaminergic neurons and the performance of olfactory tasks. Furthermore, a 100% increase of TH-positive neurons is reported in the OB tissue of Parkinson’s patients, which may be responsible for the hyposmia in these patients (38).

Most recently, study showed that patients with diabetes have lower olfactory abilities than normal cases (39). Researchers showed that the expression of GFAP protein (astrocyte cell marker) in the OB tissue and lamina propria of the olfactory epithelium decreased in the STZ-induced diabetic rats compared to the control groups (40). All these researches indicated that DM could impair OB tissue structure and function. 

On the other hand, maternal diabetes during pregnancy can have various effects on fetal development, including potential alterations in organogenesis, metabolic programming, and neurodevelopment. Gestational diabetes causes neurocognitive disorders in the offspring (41). GDM induces chronic inflammation in the offspring’s brain by increasing the activity of microglia cells (42). One study examined the olfactory function in children born to mothers with gestational diabetes. The study found that children of diabetic mothers had a higher prevalence of olfactory dysfunction compared to children of non-diabetic mothers (43). 

The exact mechanisms by which maternal diabetes may impact offspring’s olfactory function are poorly understood. It is hypothesized that the metabolic disturbances associated with maternal diabetes, such as hyperglycemia and oxidative stress, may affect the development and function of olfactory-related structures in the fetal brain. In this study, we evaluated the number of dopaminergic neurons in the OB tissue by examining the expression of TH and DDC in the OB tissue of the offspring of mothers with gestational diabetes and the control group using qRT-PCR and western blot analysis. Furthermore, the number of dopaminergic neurons was calculated using immunofluorescence for the TH marker. Our results showed that the expression of TH and DDC genes was increased in the OB tissue of male OGD at both the mRNA and protein levels. TH-positive cells are mostly characterized in the glomerular layer of the human and rat OB (44, 45). It has been estimated that nearly 10% of all juxtaglomerular cells in the GL layer of adult animals are TH-positive cells (46).

Dopaminergic periglomerular interneurons play an important role in inhibiting glutamate release from olfactory sensory fibers and controlling glomerular output information in the OB. In the present study, cell counting showed that the percentage of TH-positive cells was significantly increased in the OGD group. Therefore, according to this result, we hypothesize that the increase of TH and DDC expression in the OGD group may result from an extra supply of newborn TH-positive cells in the glomerular layer. An increase in dopaminergic activity may suppress olfaction information due to the inhibitory effect of dopamine neurotransmitters on the transmission between the olfactory receptor cells and the mitral cells within the OB tissue. Dopamine inhibits olfactory sensory fibers after binding to D2 presynaptic receptors and reduces calcium influx via N-type calcium channels (18).

## Conclusion

The present research has provided information on some changes in the OB tissue of the offspring of diabetic rats. Gestational diabetes and inappropriate uterine environment play a role in long-term disorders in adult offspring. Our findings indicated that the number of dopaminergic neurons in the OB tissue increases in the offspring of mothers with gestational diabetes. Due to dopamine being known to inhibit olfactory transmission, an increase in dopaminergic neurons in the OB tissue of OGD rats may be responsible for the olfactory dysfunction. Due to the increasing damage induced by gestational diabetes, more detailed mechanisms underlying the interactive effects of gestational diabetes on the offspring’s OB need to be explored.

## Authors’ Contributins

MJ G and Z N provided the concept and methodology, K B helped prepare the original draft, and S G provided methodology. 

## Conflicts of Interest

The authors declare that they have no known competing financial interests or personal relationships that could have appeared to influence the work reported in this paper.
